# Implications of MHC-restricted immunopeptidome in transplantation

**DOI:** 10.3389/fimmu.2024.1436233

**Published:** 2024-07-05

**Authors:** Zhuldyz Zhanzak, Davide Cina, Aileen C. Johnson, Christian P. Larsen

**Affiliations:** ^1^ Department of Surgery, Emory University School of Medicine, Atlanta, GA, United States; ^2^ Emory Transplant Center, Emory University School of Medicine, Atlanta, GA, United States

**Keywords:** transplantation, immunopeptidome, tolerance, indirect allorecognition, direct allorecognition

## Abstract

The peptide presentation by donor and recipient major histocompatibility complex (MHC) molecules is the major driver of T-cell responses in transplantation. In this review, we address an emerging area of interest, the application of immunopeptidome in transplantation, and describe the potential opportunities that exist to use peptides for targeting alloreactive T cells. The immunopeptidome, the set of peptides presented on an individual’s MHC, plays a key role in immune surveillance. In transplantation, the immunopeptidome is heavily influenced by MHC-derived peptides, delineating a key subset of the diverse peptide repertoire implicated in alloreactivity. A better understanding of the immunopeptidome in transplantation has the potential to open up new approaches to identify, characterize, longitudinally quantify, and therapeutically target donor-specific T cells and ultimately support more personalized immunotherapies to prevent rejection and promote allograft tolerance.

## Introduction

Transplantation offers life-saving therapy for end-stage organ failure. About 100,000 solid organ transplants are performed every year, thus improving the life expectancy, clinical condition, and quality of life of recipients (1). Advances in immunosuppression regimens have resulted in excellent early outcomes post-transplantation, however, long-term allograft survival rates remain suboptimal. Immune-mediated allograft injury, predominantly directed by cytotoxic CD8 T cells and helper CD4 T cells in conjunction with B cells, is a common cause of allograft failure (2). Allorecognition, the process by which T cells recognize the donor tissues, happens through two pathways, direct and indirect, depending on the antigen presentation mechanism and the presenting cell type (3). Direct allorecognition refers to the CD8 T cell recognition of either self or donor-derived peptides processed and presented by donor cells on intact donor major histocompatibility molecules (MHC). On the other hand, indirect allorecognition occurs when donor MHC molecules are processed and presented as peptides by the recipient antigen-presenting cells (APC) to recipient CD4 T cells. Despite this knowledge, methods to systematically quantify, characterize, and target donor-specific T cells associated with rejection remain an unmet need in the field.

The immune response to an allograft mainly targets mismatches between donor and recipient MHC molecules, with T cell recognition of the peptide-MHC complexes being the first step in allograft rejection (4). MHC molecules are transmembrane protein complexes that are responsible for antigen presentation to T cells. They are known to be highly polymorphic with many possible alleles at each locus resulting in significantly different proteins with specific peptide binding requirements (5). There are two major types of MHC molecules- class I and class II. MHC class I molecules predominately present peptides made from proteasomal degradation of cytosolic proteins to CD8 T cells (6). In contrast, MHC class II molecules present both intracellular and extracellular peptides, which are processed through the endosomal pathway, to CD4 T cells (7). Optimal MHC class I peptides are 8-11 amino acids in length, as these peptides sit completely enclosed in the peptide-binding groove of the MHC class I molecules (6). In contrast, MHC class II molecules typically accommodate 13-25-mer peptides with flanking regions at C and N-termini extending out of an open binding pocket, thereby leading to more conformational plasticity in antigen presentation (7). The peptide repertoire is diverse because of the vast number of proteins that are surveilled, and the variety of binding patterns required by different MHC class I and class II molecules.

The immunopeptidome is the entire set of peptides presented by an individual’s MHC molecules that enables T cell immuno-surveillance. One of the first documentations of immunopeptidome appeared in the 1980s from the Rammensee group (8). They successfully determined naturally processed 10-12-mer peptides by acid elution and by testing individual fractions for CD8 T cell recognition to identify dominant mouse MHC class I-restricted, H2-K^d^ and H2-D^b^, viral epitopes. These results demonstrated that peptide isolation can be applied to identify viral peptides recognized by CTLs (cytotoxic T lymphocytes). Similarly, the early studies of MHC class II peptides were led by Hunt and others, who eluted 16-18-mer peptides presented by mouse MHC class II, I-Ad, from cells of murine B cell lymphoma (9). This work was the first to experimentally highlight the importance of the six-residue binding motif within the MHC class II bound peptides and show that peptide binding grooves on MHC class II molecules can be open at C and N-termini. Additionally, Sette and others further discovered a novel approach to generate high-affinity MHC class II peptides by incorporating reiterative motifs with enhanced binding affinity (10). All these works cumulatively expanded our knowledge of MHC-restricted peptides. Nevertheless, our understanding of the MHC class I and class II immunopeptidomes remains incomplete largely because their complexity is compounded by the diversity of MHC allelic forms.

In this review, we first describe the MHC class I and class II immunopeptidomes and describe the role of peptides in allorecognition. We next discuss methods employing the emerging knowledge of the immunopeptidome to identify the alloreactive T cells and outline the applications of immunopeptidome in transplantation.

## MHC class I immunopeptidome and direct allorecognition

The MHC class I immunopeptidome comprises the peptide repertoire presented by MHC class I molecules that are expressed by all nucleated cells (11). MHC class I molecules are polygenic, encoding three classical human leukocyte antigen (HLA) class I genes (HLA-A, HLA-B, HLA-C) on chromosome 6 and three in mice (H2-K, H2-D, H2-L) on chromosome 17 (11–13). Additionally, they are polymorphic, encoding multiple alleles of each gene between individuals. For instance, HLA-B is known to be the most polymorphic with more than 3000 documented variants (14). MHC class I is the heterodimer consisting of heavy (α) and light chains (β2m) (7). The α chain has three extracellular domains, a transmembrane region, and a C-terminal cytoplasmic tail. The β2m is critical for MHC stability and proper folding, associating with the α3 domain (15). The α1 and α2 domains are associated with the peptide binding groove, holding MHC class I peptides of 8-11 amino acid residues in length (16). The peptide binding groove within the α1 and α2 domains is known to contain the most polymorphic residues and to determine peptide specificity (17). The preferred anchor residues within the peptide-binding groove are P2 (from the N-terminus of the peptide) and P9 (from the C-terminus), which allows for a range of peptide sequences to be accommodated (18). These MHC class I peptides are then presented to CD8 T cells by the direct allorecognition pathway.

**Figure 1:**
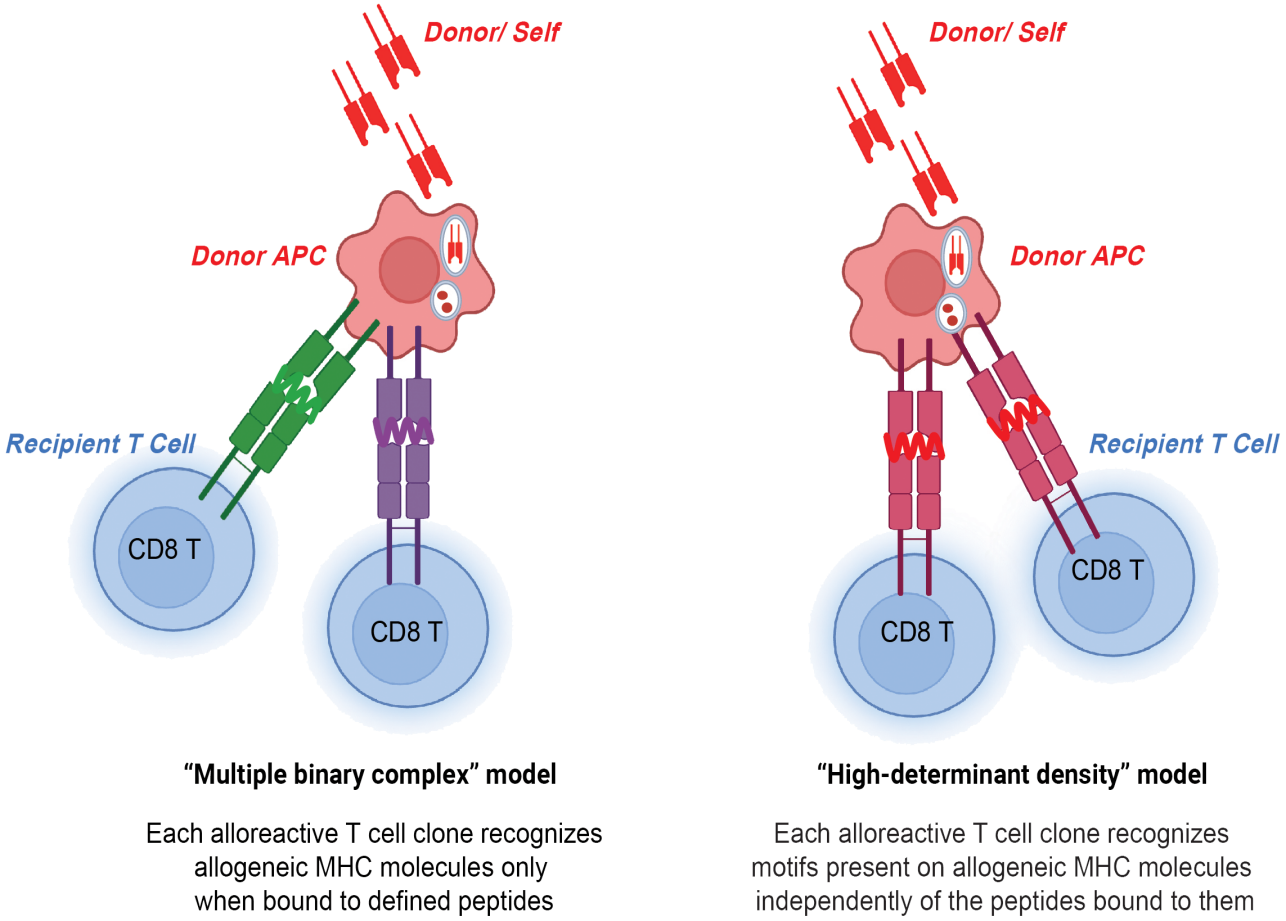
CD8 T cells in direct allorecognition. Donor and self-derived MHC class I 8-12-mer peptides are presented to recipient CD8 T cells by either peptide-dependent "multiple binary complex" model or peptide-independent "high-determinant density" model.

The direct allorecognition pathway refers to recipient CD8 T cell recognition of donor and self-derived peptides that are processed and presented by donor cells on intact donor MHC molecules (3) (**Figure 1**). There is evidence that direct alloimmune CD4 and CD8 T cell responses may be strongest early after transplantation due to the short lifespan of donor APCs (19). The mechanism of direct allorecognition by CD8 T cells was demonstrated to cause an inflammatory immune response that produces soluble cytolytic factors capable of destroying graft tissue (20). Initial sensitization of allospecific T cells in the recipient occurs mostly by direct allorecognition, followed by indirect allorecognition (21). The latter is associated with a slower response (21). The premise of direct allorecognition was first recognized in the 1960s by mixed lymphocyte reaction (MLR), in which mixtures of leukocytes from pairs of identical and nonidentical twins indicated that the changes in proliferation of leukocytes in nonidentical twins may be related to genetic differences between the two subjects (22). Later, it was shown that donor passenger leukocytes (dendritic cells) activate recipient CD8 T cells, residing predominantly in lymphoid organs, which initiate rejection in skin grafts (23). Follow-up studies by the Turka group *in vivo* revealed directly primed alloreactive T cells with a precursor frequency of 7% against a full MHC I and MHC II mismatch (24).

Two models have been proposed to explain the high precursor frequency of CD8 T cells in direct allorecognition- the peptide-independent high-determinant density model and the peptide-dependent multiple binary complex model (3). The high-determinant density model proposes that alloreactive CD8 T cells directly recognize the polymorphisms in the donor MHC class I molecule with the T cell receptor (TCR) recognizing exposed polymorphisms on the surface of the MHC class I molecule independent of a donor peptide (25). According to the multiplicity of TCR-allo-MHC interactions, even low-affinity T cells can bind MHC when ligand density is high (26). On the other hand, the multiple binary complex model postulates that each alloreactive CD8 T cell clone interacts with an MHC class I molecule bound to a defined donor peptide. The model was first introduced by Matzinger and Bevan in the 1970s and they proposed that lymphocytes reactive to major H locus respond not only to H difference but to the combination of peptide antigens together with the major H antigens (27). The importance of peptides in allorecognition was also demonstrated in human systems in which immunodominant, HLA-DR1-restricted peptide of influenza virus hemagglutinin (HA, 306-320 amino acids) could either competitively inhibit or, in some cases, augment the response of DR1-specific CD4 T cell alloreactive clones (28). Follow-up studies found that even single amino acid polymorphisms in presented peptides can govern the specificity of T cell antigen recognition (29). In the study, point mutations were introduced to the peptide sequence by substituting lysine with alanine and leucine with alanine, which resulted in a decrease and increase in TCR affinity, respectively. Overall, high-determinant density and multiple binary complex models highlight the importance of allo-MHC and allo-peptide, respectively. The high precursor frequency of CD8 T cells in direct allorecognition can be explained by these two models.

The interaction between TCR and peptide-MHC complex is important for allorecognition. The significant progress in understanding peptide presentation started with the identification of the HLA-A2 crystal structure and its antigen recognition site for CD8 T cells (30). Further studies by Garcia and others led to the first x-ray crystallography of TCR interacting with MHC class I peptide which demonstrated the central role of the complementary determining region (CDR3) of the TCR (31). They found that Vβ CDR1 and CDR2 regions were around the C-terminus locus of the class I peptide, whereas the CDR3 region was around the central position, thus suggesting the importance of the CDR3 region in peptide recognition by TCR. Later, the concept of TCR degeneracy, which is the plasticity of a single TCR to recognize multiple peptide-MHC complexes, was proposed (32). This plasticity adds to the precursor frequency of T cells during direct allorecognition (33). Given the extensive TCR degeneracy that enables recognition of a diverse range of ligands, deciphering the specific MHC class I immunopeptidome responsible for activating alloreactive CD8 T cells is crucial for the development of targeted cellular immunotherapy in transplantation. Therefore, further understanding the factors that influence which peptides are immunogenic will be important in assessing the strength of alloimmunity and developing antigen-specific tolerization approaches.

## MHC class II immunopeptidome and indirect allorecognition

Unlike MHC class I, in homeostatic conditions, MHC class II is predominantly expressed on professional APCs such as dendritic cells, B cells, monocytes, and macrophages (34), primarily presenting peptides derived from exogenous antigens internalized by these cells. In the setting of inflammation, MHC class II expression can also be induced in various other cells (35). The antigen processing machinery of these non-professional APCs, including epithelial cells, endothelial cells, and certain activated or infected cells, allows them to present peptides to CD4 T cells, thereby broadening the spectrum of antigens presented during immune responses. There are three common classical human MHC class II molecules (HLA-DR, HLA-DQ, HLA-DP) and two in mice (I-A, I-E) (7). Heterodimer MHC class II molecules consist of two α helices and two β-pleated sheets, which have the same conformation and co-localize together on chromosome 6 (36). Sequences of the α chain are less variable compared to the β chain (36). Additionally, both humans and mice encode non-classical MHC class II molecules—HLA-DM and HLA-DO in humans, and I-M and I-O in mice that facilitate the loading of peptides (37). Polymorphisms at classical MHC class I and II loci introduce variable mismatches between donor and recipient MHC molecules. Peptides derived from these polymorphic regions can be presented to CD4 T cells via the indirect allorecognition pathway.

**Figure 2:**
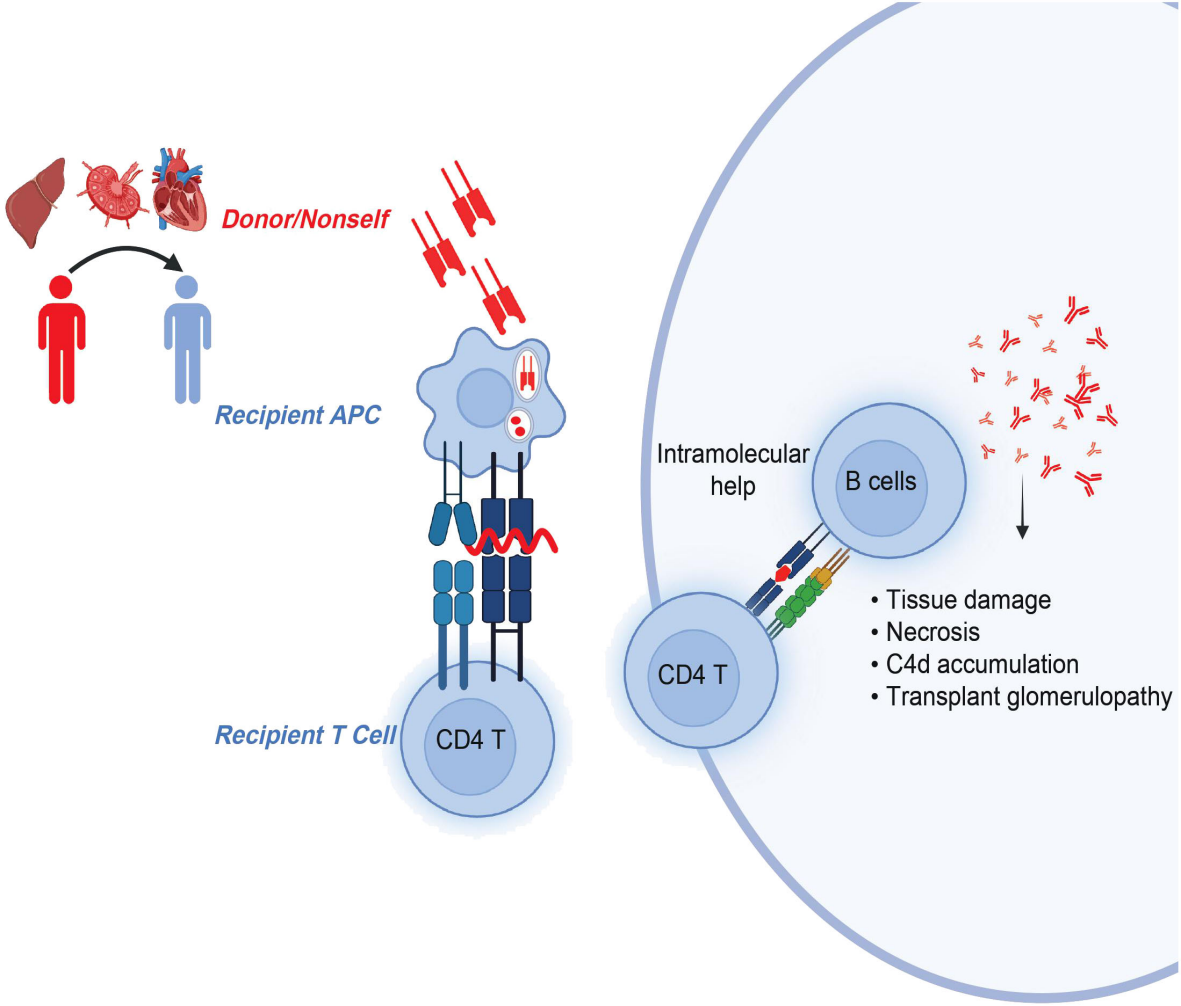
CD4 T cells in indirect allorecognition. Donor-MHC derived, polymorphic overlapping 15-20-mer peptides with flanking residues are presented by self-MHC class II molecules to recipient CD4 T cells. Activated and antigen-experienced recipient CD4 T cells further provide intramolecular help to B cells in the generation of pathogenic donor-specific antibodies.

The indirect allorecognition pathway refers to the recognition of any polymorphic and donor MHC-derived peptides by recipient T cells in the context of recipient MHC. These peptides are processed and presented by self-MHC class II molecules to self-CD4 T cells (38) (**Figure 2**). The Suciu-Foca group demonstrated that peptides presented in humans by self-HLA-DR molecule stimulated recipient T cell proliferative response, thereby indicating those cells may participate in indirect allorecognition (39). A follow-up study by Auchincloss and others found that indirect CD4 T cells play a central role as helper T cells for alloantibody production and are important for a class switch from IgM to high-affinity IgG-producing B cells (40). These findings highlight the significance of indirect CD4 T cells and their cognate peptides in rejection.

The MHC class II immunopeptidome is mainly composed of longer peptides as the MHC class II binding groove is more open due to conformational plasticity (41). The antigenic peptides presented by MHC class II molecules are mostly derived from extracellular proteins degraded by the endosomal pathway (42). The MHC class II presentation pathway starts in the endoplasmic reticulum (ER), where unfolded MHC class II molecules are prevented from binding to self and misfolded peptides by the invariant chain, Ii (43, 44). The Ii also functions to deliver MHC class II molecules to endosomal compartments close to proteases (45). In the endosome, Ii gets partially cleaved by the mix of proteases but CLIP (class II-associated invariant-chain peptide) remains bound to the MHC class II, thus blocking the binding of peptides. Once MHC class II molecules encounter the “right” high-affinity peptides, CLIP gets exchanged by the chaperone protein HLA-DM (46). HLA-DM further acts to induce a conformational change in the MHC class II molecule, thus dissociating CLIP and catalyzing peptide exchange (47). Continuous interactions with HLA-DM lead to the generation of MHC class II complexes as high-affinity peptides outcompete low-affinity peptides. Following peptide binding, newly synthesized MHC class II-peptide complexes are transported to the cell surface in vesicles and get presented to CD4 T cells (48). During this presentation, peptides get anchored into the MHC class II binding pockets at specific positions in the core binding sequence with peptide flanking residues (PFR) extended at C and N-termini. It has been demonstrated that the presence of anchor residues is required for high-affinity binding of MHC class II peptides and it occurs when anchors optimally fit within the groove (49). For HLA-DRB1, anchor residues are known to be at positions P1, P4, P6, P7, and P9, which play a crucial role in peptide binding to the MHC molecule (50). While anchor residues determine the ability of a peptide to bind, the entire core binding sequence is important in determining TCR-peptide specificity. Therefore, it is essential to further understand the properties of MHC class II immunopeptidome such as binding motifs and anchor residues since these properties will define the specificity of alloreactive CD4 T cells in transplantation.

## Immunopeptidome-based biomarkers in transplantation

The rich genomic diversity of human HLA genes is central to the alloimmune response in transplantation and indeed, better HLA matching has been shown to improve outcomes in solid organ transplantation (51). While the diversity of HLA genes is broad, not every coding or non-coding variant results in significant changes to the amino acid sequence, structure, or immune potential of a given HLA molecule. Identifying HLA mismatches that are clinically relevant to the alloimmune response is therefore a major focus of the field. Furthermore, discovering the specific HLA peptide sequences that drive immunogenicity will be important in developing immunopeptidome-based therapeutics to promote tolerance.

One major approach to cataloging clinically relevant immunogenic HLA epitopes focuses on the B-cell alloimmune response. Duquesnoy group developed a program called HLAMatchmaker that uses an *in silico* approach leveraging protein sequence, crystal structure, and stereochemical protein modeling to predict the extracellular targets of anti-HLA antibodies (52). This algorithm identifies groups of 3 adjacent, polymorphic amino acids termed “eplets” which are the minimum element that can direct antibody specificity. These amino acids do not necessarily have to be sequential but must lie within 3.0 to 3.5 Angstroms of each other on the protein surface (53). The three types of HLA class I bear considerable structural similarity and share eplets so they are evaluated together by HLAMatchmaker, while HLA class II are more distinct and therefore are not (53). The software generates lists of donor and recipient eplets based on high-resolution HLA typing and then catalogs eplet mismatches to give a more granular view of immune risk. The algorithm, however, makes several basic assumptions. The first is that all predicted eplets are target antigens for an antibody response. While there is a considerable body of literature that attempts to validate these eplets empirically using enzyme-linked immunosorbent assay (ELISA), solid phase single-antigen beads, or monoclonal antibodies, the sheer scale of the problem is such that many eplets are unverified (54, 55). HLAMatchmaker annotates eplets as ‘verified’ or ‘not-verified’. The second key assumption is that all eplets can stimulate an equally strong immune response. Tools to gauge the immunogenicity of a given eplet are somewhat lacking. One approach to this problem is to use real-world data and calculate the donor-specific antibody (DSA) event rate and event enrichment for a given eplet mismatch. However, due to linkage disequilibrium between eplets, this approach can falsely associate a given eplet with an immune outcome. Weighted correlation network analysis can overcome this by creating eplet families associated with the evolution of DSA thereby better quantifying immunogenicity (56).

Despite these limitations, eplet mismatch has been closely linked to outcomes in renal transplantation. A recent study conducted by Senev and others showed that antibody-verified eplet mismatches at the HLA-DQ locus are associated with *de novo* DSA, rejection, decreased graft function, and graft loss in a retrospective cohort of 926 renal transplant pairs with high-resolution HLA class I and class II typing (57). Other retrospective studies have shown a role for eplet mismatch at the HLA-DR locus in addition to the HLA-DQ locus in predicting *de novo* DSA formation and graft loss (58–61). This association also holds for patients on Belatacept-based immunosuppression which is associated with a lower rate of DSA formation (56). Further prospective work is needed to confirm these associations.

Predicted Indirectly ReCognizable HLA Epitopes (PIRCHE)-II is a more recently developed approach to identifying immunogenic HLA epitopes that focuses more proximally on the evolution of humoral alloimmunity on indirect T cell allorecognition. PIRCHE-II is an *in silico* method that uses the NetMHCpan algorithm to predict linear T cell antigens by modeling the binding strength of disparate donor HLA peptides to recipient HLA class II molecules as a surrogate for the potential to stimulate an indirect T cell response (62). Donor HLA-derived peptides capable of binding recipient HLA class II are segregated into self and non-self peptides. Self-peptides are then eliminated since recipient T cells are educated to be tolerant to self-peptides in the thymus. To be considered non-self, the peptides must differ from recipient peptides by at least one amino acid (63). In a way, PIRCHE-II captures the greater complexity of the evolving alloimmune response by allowing for a mismatched donor antigen to evoke a different immune response in recipients based on the recipient’s ability to present the relevant peptide on their HLA class II (64).

Much like HLAMatchmaker, PIRCHE-II suffers from several problems. First, it does not factor in how HLA antigens are processed before presentation, thereby likely overestimating the number of potential PIRCHE-II peptides (62). Second, PIRCHE-II is a theoretical framework, and the predicted peptides have not been validated empirically to stimulate an immune response. Last, by filtering out all recipient self-peptides, PIRCHE-II does not consider the possibility of cryptic recipient antigens that can stimulate a T cell response in a context-dependent manner. This being said, the relevance of PIRCHE-II is, at least in part, validated by its correlation with transplant outcomes including T-cell-mediated rejection, antibody formation, and graft survival in renal transplant recipients (57, 65, 66). While the majority of work has been done in renal transplant patients, there is also some early data in hematopoietic stem cell transplant patients suggesting that PIRCHE-II scores are inversely related to overall survival (67).

Regardless of the chosen method, these computational tools are very promising means of risk-stratifying transplant patients for a personalized approach to prognostication and immunosuppression management. Furthermore, using these tools to identify clinically relevant HLA antigenic peptides will be important for developing immunopeptidome-based immunotherapy.

## Immunopeptidome-based immunotherapy in transplantation

Achieving stable and robust immune tolerance is an important goal in clinical transplantation. Broad non-selective immunosuppressive regimens greatly reduce rates of rejection by indiscriminately targeting all alloreactive T cell populations. While effective in preventing rejection, this method suppresses tolerogenic T-cell types and also makes patients vulnerable to infections, pathogens, and malignancy (68). The goal of immunopeptidome-based immunotherapy is to specifically target injurious alloreactive T cells while preserving cell types that protect against pathogens to establish robust long-term donor-specific tolerance without immunocompromise.

Traditionally, low-throughput methods have been used to map immunogenic peptide sequences on individual MHC loci making it challenging to generalize and scale immunopeptidomics for therapeutic purposes. As computational tools for immunologic risk stratification mature, they may offer a means to streamline this process. Recently, Mohammadhassanzedeh and others used eplets derived from imputed allele level HLA data in a large SRTR cohort of 118,313 first-time kidney transplant recipients to show that of 412 potential eplet mismatches only 55 were strongly associated with death-censored graft failure (69). This not only supports the concept of a restricted number of immunodominant determinants of alloreactivity on a population scale but also the utility of computational methods for narrowing the field of practical candidate immune-therapeutic targets. As high-resolution HLA typing becomes readily available for clinical transplantation, there is an exciting opportunity for further work in this space.

While there is room for optimization in selecting targets for these emerging immunopeptidome-based therapeutics, the tolerogenic effect of exposure to appropriately selected and administered donor antigens has been established. This effect is well described in a natural setting through fetomaternal tolerance and the tolerogenic effect of non-inherited maternal antigens (NIMA). Human pregnancy involves the bidirectional exchange of fetal and maternal cells, and by association antigens across the placental barrier (70). Highly sensitive PCR for non-shared maternal DNA suggests that in a portion of healthy people, microchimerism persists well into adulthood (71). While subtle, these findings led to the theory that this microchimerism may facilitate acceptance of an allograft bearing the same disparate maternal antigens. Indeed, in renal failure patients receiving a renal allograft from their siblings, graft survival was greater when the donor had mismatched maternal HLA antigens rather than paternal HLA antigens (72). Similarly, in non-T-cell-depleted HLA haploidentical hematopoietic stem cell transplants, patients with maternal antigen-mismatched donors, had a lower incidence of graft-versus-host disease, and treatment-related mortality than those mismatched for paternal antigens (73).

Several experimental models have attempted to similarly leverage exposure to donor antigens to induce tolerance. Sayegh and others demonstrated a reduced delayed-type hypersensitivity in LEW rats upon oral administration of synthetic MHC class II peptides by downregulating the systemic cell-mediated response and inducing antigen-specific hypo-responsiveness (74). A follow-up study confirmed that oral tolerance achieved by MHC class II peptide induction altered the Th2 response, reducing interleukin-2 (IL-2) and interferon-γ (IFN-γ) production, while the intrathymic tolerance resulted in T cell anergy and clonal deletion (75). Additionally, the Benichou group used peptide therapy to induce tolerance to the immunodominant H2-Ld 61-80 epitope in Balb/c-dm2 female mice (H2-Ld negative) and found that the offspring of pregnant mice injected with H2-Ld 61-80 peptide had a decreased response to H2-Ld allospecific CD4 T cells (76). Likewise, a recent study by the Sharland group used the mass-spectrometry-based elution method to identify the murine MHC class I immunopeptidome, H2-Kd, and demonstrated that the administration of H2-Kd-expressing constructs led to tolerance (77). Overall, these findings highlight the importance of immunopeptidome in antigen identification to selectively target antigen-specific T cells and potentially induce long-term T-cell-mediated tolerance.

## Concluding remarks

Currently, long-term allograft survival post-transplantation remains a challenge. Peptide presentation either by donor or recipient MHC molecules activates T cell response which leads to allograft rejection. Characterization of the MHC immunopeptidome by identifying the specific peptide repertoire being presented can help us to better characterize the T cells involved in rejection. Besides their application in research, peptides can be developed for use in therapeutics, whether by oral tolerance, coupled to nanoparticles, expressed by vectors, or other methods. In the long run, antigen-specific tolerance, achieved by targeting those immunodominant peptides involved in alloimmunity, may open new avenues to improve transplant outcomes by more precisely targeting donor-reactive T cells, therefore avoiding many of the negative side effects of nonspecific immunosuppression. In the future, more work is required to better characterize the immunopeptidome and further advance its application in these cross-disciplinary fields.

## Author contributions

ZZ: Conceptualization, Writing – original draft, Writing – review & editing. DC: Writing – original draft, Writing – review & editing. AJ: Writing – original draft, Writing – review & editing. CL: Writing – original draft, Writing – review & editing, Supervision.
